# The relationship among pregnancy-related anxiety, perceived social support, family function and resilience in Chinese pregnant women: a structural equation modeling analysis

**DOI:** 10.1186/s12905-022-02145-7

**Published:** 2022-12-26

**Authors:** Jingui Huang, Lingli Xu, Zhen Xu, Yexin Luo, Bizhen Liao, Yan Li, Yumei Shi

**Affiliations:** 1 Department of Medical Oncology, Chongqing University Cancer Hospital, No.181 Hanyu Road, Shapingba District, Chongqing, 400030 China; 2 Department of Human Resources, Chongqing University Cancer Hospital, Chongqing, 400030 China; 3 Department of Obstetrics, The First Affiliated Hospital of Chongqing Medical University, Chongqing, 400016 China

**Keywords:** Pregnancy-related anxiety, Perceived social support, Family function, Resilience, Mediating effect

## Abstract

**Background:**

Accumulating evidence suggests that pregnancy-related anxiety (PRA) has adverse impacts on maternity health and infant development. A substantial body of literature has documented the important influence of family function, perceived social support and resilience on PRA. However, research identifying the mediating mechanisms underlying this relationship in China are still lacking. Therefore, the current study aimed to investigate the prevalence of PRA under the three-child policy in China, and also explore the interrelationships among perceived social support, family function, resilience, and PRA.

**Methods:**

In this cross-sectional study, a convenient sampling method was used to select 579 pregnant women who underwent prenatal examination at the maternity outpatient departments of the First Affiliated Hospital of Chongqing Medical University in China from December 2021 to April 2022. Participants were required to complete the following questionnaires: the demographic form, the Chinese Pregnancy-related Anxiety scale, the 10-item Connor-Davidson Resilience Scale, the APGAR Family Care Index Scale, and Multidimensional Scale of Perceived Social Support. Pearson correlation analysis was utilized to examine the rudimentary relationship among the study variables. Bootstrapping analyses in the structural equation modeling were applied to identify the significance of indirect effects.

**Results:**

There were 41.4% of pregnant Chinese women indicating PRA. Correlational analyses indicated that perceived social support, family function and resilience were negatively associated with PRA (*r* = − 0.47, *P* < 0.01; *r* = − 0.43, *P* < 0.01; *r* = − 0.37, *P* < 0.01, respectively). The results of bootstrapping analyses demonstrated significant indirect effects of perceived social support (β = − 0.098, 95% *CI* [− 0.184, − 0.021]) and family function (β = − 0.049, 95% *CI* [− 0.103, − 0.011]) on PRA via resilience.

**Conclusions:**

Chinese pregnant women are suffering from high levels of PRA. Better family function and perceived social support might reduce the occurrence of PRA, as well as by the mediating effects of resilience. Healthcare providers must be concerned about PRA and perform corresponding actions to reduce it. By strengthening social support and improving family function, antenatal care providers could effectively reduce or prevent PRA. And more importantly, implementing resilience-promoting measures are also essential to relieve anxiety and support mental health in pregnant women.

## Introduction

Pregnancy-related anxiety (PRA) refers to a kind of distinct anxiety or worries particular to pregnancy (e.g., baby’s health, mother’s health, childbirth fear, and future parenting concerns) [1]. Anxiety during pregnancy was regarded as the potent psychological predictor of child birth and development and to be independent of generalized anxiety [2]. Compared with general anxiety, PRA may correlate more strongly with negative maternal and childbirth outcomes such as hypertension, low birth weight, spontaneous abortion and preterm birth, and predict these outcomes more reliable and accurate [2–4]. In addition, severe PRA has been identified to be concerned with attention-deficit hyperactivity disorder, negative emotion and development retardation of children [5, 6]. Therefore, untreated PRA not only negatively affect women’s physical and psychological health but also serious harm of fetus’ health.

The worse thing is that PRA is a widespread problem among pregnant women during pregnancy. The prevalence of PRA accounted from 23.6 to 55% in developing countries [7–11], and the magnitude of PRA in developing countries was estimated to be 6% to 29% in international studies [12–14]. In China, the incidence of PRA was about 21 − 30% under the one-child policy before 2016 [15], and 29 − 32% under the two-child policy before May 2021 [16, 17]. And in the place of current study, Chongqing municipality, the epidemiology of prenatal anxiety was 15% at early-pregnancy [18]. Significantly, China shifted from the two-child policy to the three-child policy by allowing all couples to have up to three children on 31 May 2021 [19]. In particular, Chinese families have a long-standing preference for sons over daughters in Chinese culture [20]. Under the three-child policy, couples with strong son preference may continue to have more children until they have the desired number of boys in the family, which may potentially increase the risk of anxiety during pregnancy. Currently, there is a lack of studies on PRA among the pregnant women after the introduction of the three-child policy in China, let alone Chongqing municipality.

Previous studies reported the factors that affect PRA among pregnant women include age, education level, parity, gestational week, economic status and partner relationship [16, 21–24]. In addition, it has been established that perceived social support and family function are all important protective factors for PRA [25, 26]. Perceived social support, reflecting individuals’ true and subjective feelings, is defined as ‘individuals’ perception of the support that they receive from their social network’, which can enhance their confidence to solve diverse problems more effectively [27]. On the basis of the psychological stress theory, social support plays a major role as a buffer mechanism when people suffer from stressful events and can promote their physical and mental well-being [28]. Family function refers to the degree to which a family performs as a unit to manage activities, conditions, external stimuli, or events that cause stress with external events, which plays a significant part in personal development and social progress [29]. Family function is more crucial for pregnant women in the families from Asia and other countries with a Confucian cultural context [30, 31]. A cross-sectional study in China found that the risk of depression symptoms in pregnant women who had poor family function was 3.67 times as much as that in the better functions group [32].

Apart from perceived social support and family function, a number of studies have justified the fact of resilience in mitigating anxiety during pregnancy [25, 33]. Resilience is the capability to withstand or recover from trauma, threats, adversity, and other significant sources of stressors [34]. Individuals with higher resilience have better psychological adjustment and cope effectively when dealing with periods of intense stress [35]. Previous studies have testified that more resilient nurses are better equipped to deal with obstacles, adverse experiences, and decrease their burnout [36]. Moreover, resilience mediated the relationship between unpleasant conditions and mental health status [37, 38]. The three factors interact with each other to complicate the anxiety during pregnancy. However, little is known about the internal relationships between family function, perceived social support, resilience, and PRA among pregnant women. Previous reports pointed out that external conditions such as social support, family support, and other supports are identified component of resilience resources that can contribute to it [39, 40]. A study of quality of life among migrant older adults found resilience played a mediating role between perceived social support and mental quality of life [41]. So, we speculate that resilience in pregnant women may also regulate the relationship between family function, perceived social support and PRA.

The Healthy China 2030 Plan requires that healthcare system should screen, evaluate and manage the pregnancy-related risk factors for pregnant women, and ensure maternal and newborn safety [42]. The health facilities did not implement PRA screening program yet in China, meaning that health workers are missing PRA cases under the three-child policy. So, there is an urgent need to investigate PRA among Chinese pregnant women after the transition from the two-child policy to the universal three-child policy in 2021. More importantly, despite a body of evidence showing the connection of PRA with perceived social support, family function and resilience independently, research probing into the relationship that might exist between the four variables is scarce. With due consideration of the analysis above, the first purpose of the current research is to explore the prevalence of PRA among Chinese pregnant women, and fill current gaps in knowledge about the level of PRA under the three-child policy. And the second purpose is to examine the interrelationships among perceived social support, family function, resilience, and PRA via a structural equation modeling analysis. The results of this study could be helpful in offering new insights into analysis of the relationship between the four variables, and also empirical supports and scientific evidence for certain tailored strategies toward at mitigating the risk of PRA.

### Present research

**Fig. 1:**
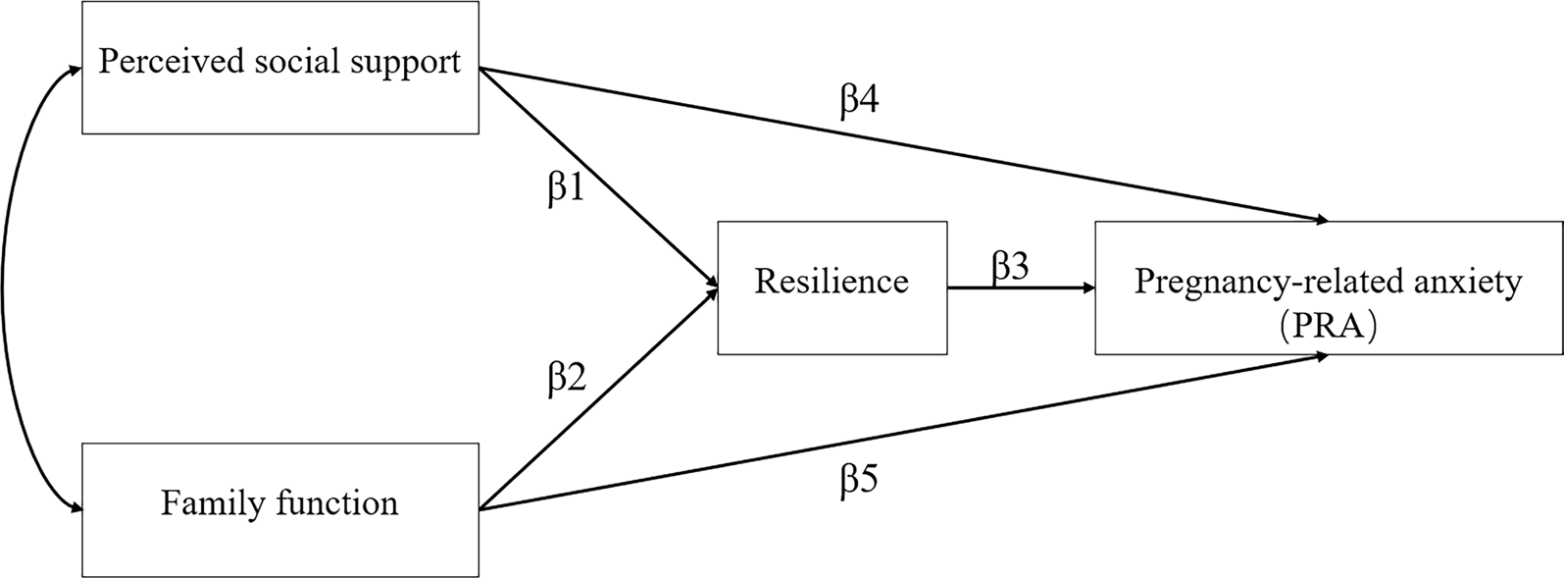
A conceptual diagram of the hypothesized mediation model

On the basis of literature review, we hypothesized a mediator model as shown in Fig. 1, including the following paths: (1) Direct paths: Perceived social support affects resilience positively (β1); Family function affects resilience positively (β2); Resilience affects PRA negatively (β3); Perceived social support affects PRA negatively (β4); Family function affects PRA negatively (β5). (2) Indirect paths: Resilience serves as a mediating role between perceived social support and PRA (β1β3); Resilience plays a mediating role between family function and PRA (β2β3).

## Material and methods

### Study setting and design

This is a hospital-based cross-sectional survey. The participants were recruited using convenience sampling method from the maternity outpatient departments of the First Affiliated Hospital of Chongqing Medical University in China from December 2021 to April 2022. Three investigators of our authors, fully trained and certified, used a face-to-face survey to collect the data after signed informed consent was obtained. Specific procedure is as follows: When pregnant women established the health records (gestational week ≥ 11) or attended general maternity examination at Obstetric clinic, investigators would review their health records. Pregnant women who were accorded with the eligibility criteria would be invited to participate in this survey. Then participants filled in the anonymous questionnaires by themselves. For those who did not have a good understanding of the questionnaire content, the research personnel would give corresponding and succinct interpretation instead of biased or instructive suggestions. The investigators were requested to check every questionnaire for accuracy and completeness on the spot. An ethical approval was obtained from the First Affiliated Hospital of Chongqing Medical University for conducting this study.

### Sample size calculation

First, this study is to investigate the prevalence of pregnancy-related anxiety. According to the previous literature, the prevalence of pregnancy-related anxiety in Chinese pregnant women was about 30% [16].With a confidence interval of 95% and 4% tolerable margin of error (*d* = 4%), the calculated sample size is* n* =${\left(\frac{{Z}_{1-\alpha /2}}{d}\right)}^{2}p(1-p)$  = 504. Then, adding a non-response rate of 10%. Thus, the total sample size is 560.

Second, another purpose of our study is to examine the interrelationships among perceived social support, family function, resilience, and PRA via a structural equation modeling analysis. The minimum sample size requirement of the structural equation was 200 [43]. To sum up, the final sample size required in this study is 560.

### Target population

The target population of this research is the pregnant women who underwent prenatal examination at the maternity outpatient departments. Pregnant women were eligible to participate in the study if they met the following criteria: aged 18 or above, singleton pregnancy, being able to read and write Chinese, and volunteering to take part in the survey. The exclusion criteria comprised: age less than 18 years; with pregnancy complications (e.g. gestational diabetes mellitus, hypertension, pre-eclampsia), as well as diagnosed and/or being treated for anxiety or depression; fetal disorders and abnormality.

### Variables and measure scales

#### Demographic form

Information on demographic characteristics was gathered including age, education level, working status, district, monthly income (yuan), trimester, parity, planned pregnancy and prior abortion.

#### Chinese pregnancy-related anxiety scale (C-PRA)

The PRA was assessed by the Chinese Pregnancy-related Anxiety scale (C-PRA). C-PRA consists of 13 self-rated items, across three dimensions: ① anxiety on self-care (6 items: the worries on how pregnancy would change their life, such as concerns about fetus’s sex not satisfying expectations from the family, worries about loss of attractiveness towards husband, worries against how pregnancy would affect their appearance or daily work); ② anxiety related to the health of the fetus (5 items: the worries about the development of fetal and whether their lifestyle such as diet would influence fetal health or not); ③ fear of childbirth (2 items: concerns about labor pain or possible adverse consequence during labor.) [44]. Each item scores 1–4 with 1 meaning never worry, and 2 always worry, with a total score of 13–52. Higher the total score is, higher level of PRA the women have. And when someone’s score ≥ the 75th percentile (≥ 24 scores), she will be evaluated as PRA [23, 44]. The test–retest reliability coefficient and Cronbach’s α coefficient was 0.79 and 0.81 respectively [44]. The Cronbach's α was 0.825 in the current study.

#### APGAR family care index scale (APGAR)

Family APGAR originally created by Smilkstein [45] was adopted to measure family function, which was an assessment tool to assess an family member’s subjective satisfaction of family functions. The APGAR is composed of 5 items (partnership, adaptability, affection, growth and resolve.) and scored on a Likert-type format with 3 options (0 = hardly ever, 2 = almost always). The total score varies between 0 and 10, with higher scores representing better family function. The APGAR has since been adopted by many Chinese scholars with good internal reliability and validity [46–48]. The Cronbach's α was 0.879 in the current study.

#### Connor-davidson resilience scale (CD-RISC)

The resilience of pregnant women was tested by the 10-item Connor-Davidson Resilience Scale (CD-RISC-10). Wang and his colleagues translated it into Chinese with excellent validity and reliability (Cronbach’s α = 0.91) [49]. The responses of each item are scored on a Likert-type format with 5 options (0 = never, 4 = always). The total score ranges from 0 to 40 with higher scores denoting greater resilience. The Cronbach’s α of this whole scale was 0.91 for our study.

#### Multidimensional scale of perceived social support (MSPSS)

The 12-item Multidimensional Scale of Perceived Social Support (MSPSS) was adopted to measure perceived social support (family, friends, and significant others). Zimet et al. [50] created the original English version of the MSPSS and reported high internal consistency among 265 pregnant women. Huang and Jiang [51] translated it into Chinese and modified it. Participants were asked to fill out the 7-point scale ranging from 1 (strongly disagree) to 7 (strongly agree). Higher total scores are indicative of a higher level of perceived social support. In the present study, the Cronbach’s α for MSPSS was 0.92.

### Statistical analysis

Epi Data version 3.1 and SPSS version 25 were used to record and analyze data. Categorical data and continuous data were listed as frequency and percentage, and mean and standard deviation (SD), respectively. Harman's single-factor test was used to detect the common method bias. Variance inflation factor (VIF) and Cook’s distances were used to test the multicollinearity and outliers, respectively. The relationships between variables was established using Pearson correlation analysis. Statistical significance level was considered as a two-tailed *P* -value of value less than 0.05.

We implemented a structural equation modeling (SEM) with observed variables using the Maximum Likelihood estimation method to examine the theoretical model hypothesis. Bootstrapping method of repeat sampling by 2000 times was applied to calculate the 95% confidence interval (*CI*) for the indirect effect, and the indirect effect is considered significant if 95% *CI* without containing zero. SEM was conducted using AMOS 23.0. The SEM model was assessed using the following indexes and recommended limits: χ^2^/*df* < 3; tucker lewis index, TLI > 0.90; normed fit index, NFI > 0.90; root mean square error of approximation, RMSEA < 0.08; goodness of fit index, GFI > 0.90; incremental fit index, IFI > 0.90; comparative fit index, CFI > 0.90 [52].

## Results

### Common method bias, multicollinearity and outliers

Common method bias (CMB), multicollinearity and outliers should be reviewed before proceeding. A cross-sectional design and self-reported data might lead to CMB [53]. So, we adopted the Harman's single-factor test to detect it, which is widely used in common method bias testing. After the principal component factor analysis, we extracted 8 eigenvalues greater than 1. And the variance explained by the first factor was only 29.47%, which was less than 40% of the critical standard [54]. Thus, no substantial CMB problem exists in the current study. VIF (< 5.0) and Cook’s distances (< 1.0) were computed to identify the potential existence of multicollinearity and outliers, respectively. Results indicated that there were no problem of outliers and serious multicollinearity in this study (VIF 1.401–1.612; Cook’s distances 0.000–0.883).

### Characteristics of the participants

Among the 579 eligible participants who volunteered to take part in the study, 18 participants failed to finish the questionnaires for lack of time or other personal reasons. Thus, 561 participants completed the questionnaires with a response rate of 96.9%. Among them, the age of the participants ranged from18 to 42 years old, with a mean age of 29.04 ± 3.78 years. Almost four-fifths (82.5%) of the study participants completed a college education. Over One hundred and eighty (82.2%) of them belonged to employed status. As for obstetric characteristics, the mean gestational age of the respondents was 29.66 ± 9.45 weeks, and 379 respondents (67.6%) were in their third trimester. Other basic information is displayed in Table 1.

**Table 1:** Demographics of the participants

Variate	Category	*N* (%)
Age (years)	18–24	47 (8.4)
	25–34	468 (83.4)
	35–42	46 (8.2)
Education level	High school or below	98 (17.5)
	Undergraduate college or above	463 (82.5)
Working status	Employed	461 (82.2)
	Unemployed	100 (17.8)
Districts	Urban	507 (90.4)
	Rural	54 (9.6)
Monthly income (yuan)	≤ 8000	328 (58.5)
	> 8000	233 (41.5)
Trimester	First	50 (8.9)
	Second	132 (23.5)
	Third	379 (67.6)
First pregnancy	Yes	341 (60.8)
	No	220 (39.2)
Planned pregnancy	Yes	392 (69.9)
	No	169 (30.1)
Prior abortion	Yes	131 (23.4)
	No	430 (76.6)
Conception type	Spontaneous fertilization	534 (95.2)
	Assisted fertilization	27 (4.8)

		Mean (SD)
Age	**–**	29.04 (3.78)
Gestational week	**–**	29.66 (9.45)

*SD* standard deviation

### The correlation between perceived social support, family function, resilience and PRA

The proportion of pregnant women with positive screening results for pregnancy-related anxiety symptoms (C-PRA ≥ 24) was 41.4% (*n* = 232). We calculated the mean value, SD, and correlation coefficients of each variable, and the results are showed in Table 2. Table 2 showed a positive relationship between perceived social support with resilience (r = 0.58, *P* < 0.01) and family function with resilience (r = 0.47, *P* < 0.01), and analysis also indicated a negative association between perceived social support and PRA (r =  − 0.47, *P* < 0.01), resilience and PRA (r =  − 0.43, *P* < 0.01), and family function with PRA (r =  − 0.37, *P* < 0.01).This preliminarily indicated the relationship between the four variables of Chinese pregnant women, which offered rudimentary support to the hypothetical paths mentioned earlier.

**Table 2:** Correlation analysis between variables

Variables	1	2	3	4
1.MSPSS (range: 35–84)	1	–	–	–
2.CD-RISC-10 (range: 7–40)	0.575**	1	–	–
3.APGAR (range: 1–10)	0.480**	0.469**	1	–
4.C-PRA (range: 13–46)	− 0.470**	− 0.433**	− 0.371**	1
Mean	64.83	26.56	8.15	23.42
SD	8.71	5.76	1.92	5.58

^**^P < 0.01

*MSPSS* Multidimensional Scale of Perceived Social Support; *CD-RISC-10* the 10-item Connor-Davidson Resilience Scale; *APGAR* APGAR Family Care Index Scale; *C-PRA* Chinese Pregnancy-related Anxiety scale; *SD* standard deviation

### Mediating effect of resilience

**Fig. 2:**
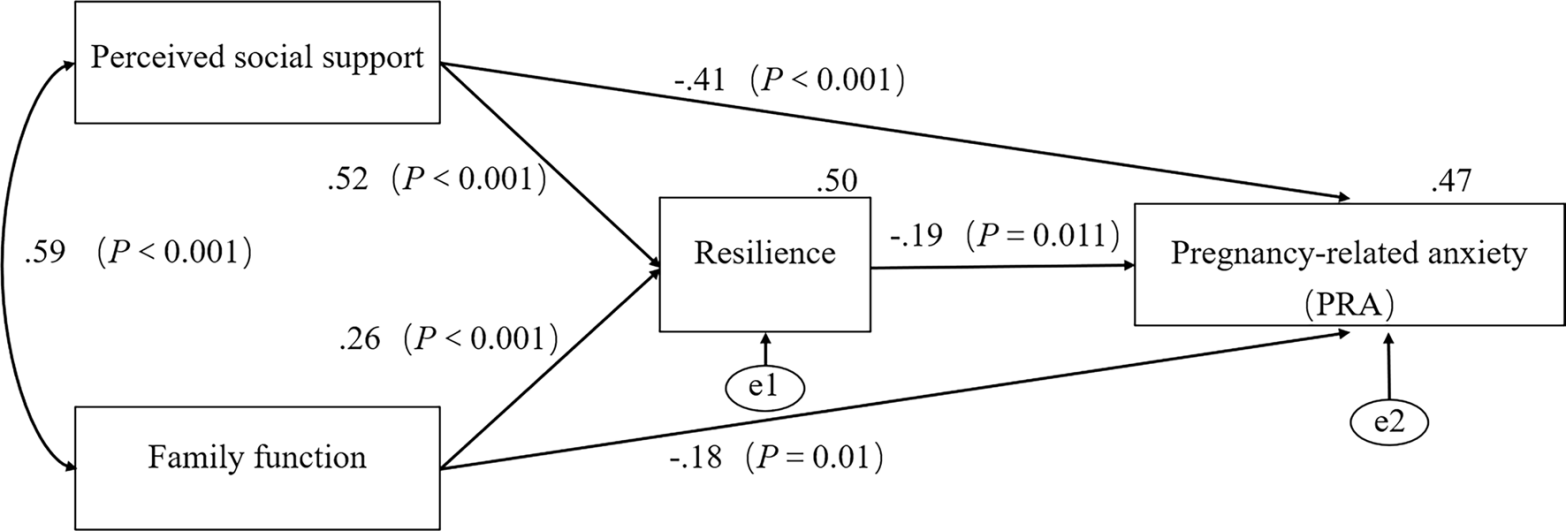
The standardized path coefficients in mediation analyses

The standardized coefficients for all pathways in the mediator model of resilience were shown in Fig. 2. The following indicators showed that the hypothetical model fit the data well: χ^2^/*df* = 1.987, TLI = 0.958, NFI = 0.930, RMSEA = 0.042, GFI = 0.941, IFI = 0.964, CFI = 0.964.

Based on the model, the standardized coefficient of perceived social support, family function, and resilience to PRA was − 0.41, − 0.18 and − 0.19, respectively. And the results of the model indicate that all direct paths were significant (*P* < 0.05). We applied the “bootstrapping method” repeated sampling 2,000 times, and calculated 95% *CI* to test whether resilience should be considered as a mediator for the association among perceived social support and family function with PRA. As proposed, the indirect path of perceived social support on PRA (β1β3 = − 0.098, 95% *CI* [− 0.184, − 0.021]) and family function on PRA (β2β3 = − 0.049, 95% *CI* [− 0.103, − 0.011]) through resilience was significant because the confidence interval did not include zero. The results are presented in Table 3.

**Table 3:** The direct and indirect effects of pathways

Effect	Standardized pathway	SE	95% CI		*P* Value
			Lower	Upper	
*Direct effect*					
β1: Perceived social support → resilience	0.52	0.011	0.409	0.626	< 0.001
β2: Family function → resilience	0.26	0.088	0.141	0.371	< 0.001
β3: Resilience → PRA	− 0.19	0.195	− 0.332	− 0.029	0.011
β4: Perceived social support → PRA	− 0.41	0.038	− 0.576	− 0.243	< 0.001
β5: Family function → PRA	− 0.18	0.290	− 0.353	− 0.015	0.01
*Indirect effect*					
β1β3: Perceived social support → resilience → PRA	− 0.098	0.041	− 0.184	− 0.021	0.017
β2β3: Family function → resilience → PRA	− 0.049	0.023	− 0.103	− 0.011	0.012

*PRA* pregnancy-related anxiety; *CI* confidence interval; *SE* standard error

## Discussion

### The prevalence of PRA

We investigated the prevalence of PRA in Chinese pregnant women and explored the mediating role of resilience. PRA occurred in 41.4% of pregnancies in our study, which is similar to the reported prevalence in south-west Nigeria (43.5%) [10]. And the level of PRA yielded by us is much higher than that reported in the Netherlands (11%) [13], Tanzania (25%) [8], Iran (21%) [55] and Qatar (26.5%) [25], whilst it was lower than what was reported in India (55.7%) [11]. Such discrepancy in the percentages reported could be due to various measuring tools used, as well as the cultural multiplicity and sociodemographic diversity of the target population. A Chinese study showed that the prevalence of PRA was about 30% at mid-pregnancy or late-pregnancy under the two-child policy in China [16], which is much lower than that reported in the current study. For pregnant women, especially older pregnant women and multiparas, they would have more and multiple worries or pressure, such as own health, work-family conflicts, childrearing pressure and fetal gender under the three-child policy [56], which might lead to an increase of anxiety during pregnancy. Our findings suggested that the occurrence rate of PRA is high among Chinese pregnant women under the three-child policy, and maternal care providers should give full consideration to reduce the prevalence of PRA by taking effective measures.

### Direct relations

This study explored the ways by which perceived social support, family function, and resilience affected the PRA of pregnant women. The results indicated that perceived social support could have a direct effect on the PRA, which is in line with the results reported by Naja in Qatar and Brunton in Australia [25, 57]. This finding can be explained in two ways. First, this might be attributed to the ‘buffering model’ whereby social support serves as a buffer between adverse life events or stress and mental health [58, 59]. The rapid growth of the fetus makes the organs of the mother closer to the maximum functional load in the third trimester. Childbirth fear, worries on the fetal health, and physical discomfort would inevitably bring psychological stress on pregnant women [60]. Adequate social support could protect individuals from deleterious effects of the above stressors, and help pregnant women keep positive emotions and reduce anxiety. Second, social support can practice the coping and assessment ability of individuals, reduce the perceived severity of events and thus play an indirect protective role in individuals’ negative emotions [61]. The association of perceived social support and PRA suggested that support from families, friends and other sources during pregnancy is a focus field for healthcare professionals to assess and manage. Pregnant schools in many hospitals, mainly reflected in maintaining health during pregnancy and in disseminating healthcare knowledge, are an major source of social support for pregnant women at present [62]. Maternity care staffs should offer formal support including psychological counselling and professional informational support to the entire family, promote the adaption of pregnancy, and reduce the unnecessary anxiety. Significantly, spirituality and spiritual care play an important part in providing effective emotional support for patients [63]. So, it is also suggested that medical staffs should raise the awareness about spirituality and spiritual care to offer more support for pregnant women.

Furthermore, our results verified that family function also had a direct impact on PRA. The study conducted by He in China is consistent with our result [64]. In a healthy family, family members can detect the psychological and physical changes of pregnant women, and provide timely material and spiritual help when women cope with anxiety or stressors [65]. However, dysfunctional families often have the characteristic of lower warmth, lower expressiveness and lower cohesion, and but also higher rigidity, conflict and affectionless control, which may be the source of anxiety [66]. Families with domestic violence against women is the reason of many mental illnesses and physical injuries [67]. It is recommended for maternity care providers to encourage family members, especially spouses, strengthen the deeper emotional communication with perinatal women to understand their inner needs of family support and then develop a detailed care plan according to the needs. Community staffs can even proceed regular family visit to ensure that family members are implementing the family support plan, identify problems in family support critically and make effective recommendations to family members.

In line with finding from prior studies [25, 57], resilience had a close negative relationship with PRA in our study. Resilience is a dynamic adaptive process in which individuals respond to adverse events eagerly and make efforts to adapt to the new role or environment [68]. Pregnant women with higher resilience would maintain mental well-being by making full use of available resources to accommodate to the significant changes and learn to cope with pregnancy-related stress or depression effectively [37, 69].

### Indirect relations

Another important new discovery of our research was that resilience mediated the effects of perceived social support and family function on PRA, respectively. Similarly, a Japanese study also found that resilience had a partial mediating effect between family function and psychological health in hemodialysis patients [70]. Besides, a Chinese research showed that resilience also significantly mediated the links between perceived social support and mental health in teachers [71]. Excellent family function and higher perceived social support can buffer the negative impacts of stressors and provide favorable external environmental conditions for the enhancement of resilience [72, 73]. In a word, greater perceived social support and better family function contributed higher resilience, thus generate positive emotions and alleviate PRA accordingly. Thankfully, resilience has become recognized less as an innate characteristic and more as a set of learnable skills [74]. Cognitive behavioral therapy, mindfulness based stress reduction, and enhancing social support and optimism are potential intervention that serve to increase an individual’s resilience at present [75–77].

The present study had three strengths. First, this is the first work to analyze the interrelationships and potential mechanisms of PRA, perceived social support, family function and resilience of pregnant women by utilizing an SEM, which may have important implications for antenatal care providers and researchers alike. Second, the current study investigated the prevalence of PRA in the context of the three-child policy in China, which provided a reference for developing specific interventions to some extent. Third, a high response rate and the accuracy of the information were guaranteed by face-to-face interviews.

### Limitations

However, a few limitations of this study also merit consideration. First, a convenience sampling method was used, and data were collected in single center, which limited the findings generalize to the whole country and may have introduced bias. Second, the cross-sectional design made it difficult to derive causal interpretations of the relationships among these variables and the claim of the mediating effects of resilience to be somewhat questionable. Third, we did not take the confounding factors into consideration in our study, such as epidemic caused by Corona Virus Disease 2019, which appear to be relevant to exacerbation of anxiety or depression in high-risk group such as pregnant women and health care workers [78–80].

## Conclusions

In brief, the results of the current study highlighted the necessity for specific antenatal screening of PRA among pregnant Chinese women in the context of the three-child policy. The present study also indicated that resilience served as a mediator between perceived social support and PRA, and family function and PRA through the examination of a quantitative model. Therefore, antenatal care providers can take the following specific measures to minimize the negative impact of PRA for perinatal women: actively mobilize the social support system, bolster family function and build their resilience.

To eliminate the limitations of this study, a wider geographical and national study is suggested to determine PRA among pregnant women in different healthcare areas during the COVID-19 pandemic. It is proposed that future research be carried out by using replications and additional longitudinal study design that focus on changes in relationships among these variables over time. Randomized sampling method is also required.

## Data Availability

The dataset generated and analyzed during the current study are not publicly available due to promises of participant anonymity and confidentiality but are available from the corresponding author on reasonable request.

## Abbreviations

PRA: Pregnancy-related anxiety
C-PRA: The Chinese Pregnancy-related Anxiety scale
CD-RISC-10: The 10-item Connor-Davidson Resilience Scale
MSPSS: Multidimensional Scale of Perceived Social Support
SD: Standard deviation
CMB: Common method bias
VIF: Variance inflation factor
SEM: Structural equation modeling
CI: Confidence interval
TLI: Tucker lewis index
NFI: Normed fit index
RMSEA: Root mean square error of approximation
GFI: Goodness of fit index
IFI: Incremental fit index
CFI: Comparative fit index
SE: Standard error
